# Effects of Melatonin and Calcium Chloride Treatment on the Quality of Chinese Kale Sprouts

**DOI:** 10.3390/foods15101772

**Published:** 2026-05-17

**Authors:** Kehao Liang, Yang Li, Pufan Zheng, Xuena Yu, Fen Zhang, Cunkun Chen, Wenjuan Cheng, Bo Sun

**Affiliations:** 1 College of Horticulture, Sichuan Agricultural University, Chengdu 611130, Chinazhangf@sicau.edu.cn (F.Z.); 2 Institute of Agricultural Products Preservation and Processing Technology, Tianjin Academy of Agricultural Sciences (National Research Center of Agricultural Products Preservation Engineering and Technology), Tianjin 300384, Chinacck0318@126.com (C.C.); 3 Key Laboratory of Storage and Preservation of Agricultural Products, Ministry of Agriculture and Rural Affairs, Tianjin Key Laboratory of Postharvest Physiology and Storage and Preservation of Agricultural Products, Tianjin 300384, China; 4 Institute of Agricultural Resources and Environment, Tianjin Academy of Agricultural Sciences, Tianjin 300384, China

**Keywords:** calcium chloride, Chinese kale, glucosinolates, melatonin, total phenolic content

## Abstract

Sprout vegetables have emerged as functional instant foods, with elevated concentrations of bioactive compounds compared with their mature counterparts. Chinese kale (*Brassica oleracea* var. *alboglabra*) is a cruciferous *Brassica* vegetable particularly rich in phenolic compounds, glucosinolates, and other nutrients, making it a suitable candidate for sprout production. This study aimed to explore the impact of melatonin (MT), calcium chloride (CaCl_2_), and their combination on the quality and functional metabolism of Chinese kale. The results showed that MT treatment alone led to significantly higher ferric-reducing antioxidant power and concentrations of chlorophylls, carotenoids, soluble sugar, soluble protein, flavonoid, total phenolic compounds, and glucosinolates than those under CaCl_2_ treatment alone. CaCl_2_ treatment alone increased ascorbic acid content by 30.5%, but had limited effects on protein accumulation and secondary metabolites. However, the combined treatment did not exert a synergistic effect on ascorbic acid content, which decreased by 19.8% compared with that under the control treatment, significantly (*p* < 0.05). Overall, MT treatment was effective in boosting nutrient levels, thereby elevating the functional quality of Chinese kale sprouts.

## 1. Introduction

Cruciferous vegetables are diverse and safe for consumption. They are widely cultivated and consumed worldwide owing to their marked health-promoting properties [1,2]. Increased public attention to food safety and enhanced awareness toward nutrition and health in recent years have further highlighted the value of cruciferous vegetables. Sprout vegetables have gradually become the representative of modern green healthy foods due to their high nutritional value, short growth cycle, and low pollution risk [3]. These vegetables are usually young edible parts produced by cultivating plant seeds or other propagules in a controlled environment, such as buds, seedlings, or tender stems. Sprout vegetables grow rapidly, are simple to cultivate and manage, and are not limited by season or region. Hence, their production is considered to be a resource-saving agricultural model with the advantages of a green economy and high efficiency [4]. Cruciferous sprouts have a brief growth cycle, high cleanliness, and rich nutrition. Also, their glucosinolate content is much higher than that in mature plants. As unique secondary metabolites of cruciferous plants, glucosinolates and their endogenous enzyme myrosinase constitute a key chemical defense system. Active substances such as isothiocyanates produced after enzymatic hydrolysis not only help plants resist biological stress but also have become an important indicator of the functional quality of these vegetables because of their anticancer activity and unique flavor [5,6,7]. The total amount of glucosinolates in sprouts can reach more than 10 times that in mature organs. For example, in Chinese kale, the content of glucosinolates in sprouts is significantly higher than that in leaves and stems [8]. Therefore, cruciferous sprouts have higher nutritional value in the human diet and thus have become an important part of a functional diet.

Melatonin (MT) is an important endogenous indoleamine hormone in plants [9]. Key developmental processes throughout the plant life cycle, from seed germination to leaf senescence, are regulated by MT. This includes the modulation of growth, photosynthetic efficiency, and aging-related pathways [10,11]. Furthermore, MT functions as a potent antioxidant, preserving cellular redox homeostasis by scavenging excess reactive oxygen species (ROS) [12]. Exogenous application of MT has been shown to maintain the visual quality of stored broccoli while enhancing glucosinolate content and antioxidant capacity [13]. Similarly, the application of 100 μmol L^−1^ MT delayed chlorophyll degradation and increased total glucosinolate content [14,15]. Moreover, MT can effectively promote the upregulation of flavonoid synthesis-related genes in black bean sprouts, thereby inducing flavonoid synthesis [16].

Calcium serves as a crucial second messenger, which is integral to cellular signal transduction and multiple plant metabolic pathways [17]. Moreover, it is an essential nutrient required for the synthesis and stability of cell walls, as well as the development of various organelles [18]. The external application of calcium chloride (CaCl_2_) exerts beneficial effects on the postharvest preservation of broccoli. However, the effectiveness of CaCl_2_ treatment alone can vary depending on the cultivar. Recent studies suggest that combining CaCl_2_ with other postharvest agents can enhance its efficacy. For instance, CaCl_2_ in combination with kojic acid effectively inhibited broccoli yellowing, retained its characteristic volatiles, and improved the quality [19]. Furthermore, CaCl_2_ treatment enhanced both the yield and antioxidant content of broccoli sprouts while also significantly boosting their glucosinolate content [20,21].

This study was conducted to investigate the influence of MT and CaCl_2_, applied singly or in combination, on the growth morphology and key nutritional components of Chinese kale sprouts, with the objective of clarifying their regulatory roles and providing a theoretical basis for improving the quality.

## 2. Materials and Methods

### 2.1. Plant Material and Treatment

Seeds of Chinese kale (“sijicutiao”) procured from Guangzhou Hongye Seed Technology Co., Ltd (Guangzhou, China). were used in the study. The purity of seeds was not less than 96%, and the germination rate was not less than 85%. Seeds of the same size were selected and divided into control (distilled water) and treatment groups (MT, CaCl_2_, and MT + CaCl_2_), with 12 g of Chinese kale seeds sown in each group. The seeds were soaked in 40 mL of distilled water at 55 °C, incubated at 37 °C for 3–4 h, and then sown on a plastic seedling tray (33 × 26 × 4.5 cm^3^) for hydroponics. The seedling trays were subjected to a dark period of 48 h in the growth chamber and then exposed to white light. The seedlings were cultured under the conditions of light intensity of 80 μmol m^−2^ s^−1^, temperature of 23 °C, and humidity of 70%. The photoperiod was 16 h of light and 8 h of darkness. From days 4 to 9, the seedlings in the control group and the three treatment groups were sprayed with distilled water, 100 μmol L^−1^ MT solution [22], 10 mmol L^−1^ CaCl_2_ solution [23], and 100 μmol L^−1^ MT solution + 10 mmol L^−1^ CaCl_2_ solution, respectively. The leaf surface was evenly covered with droplets (about 6 mL). On day 10, the sprouts were harvested by removing the roots and freeze-dried, and their nutritional indicators were measured.

### 2.2. Plant Fresh Weight and Height

On day 10, the fresh weight of the entire tray from each treatment was measured. Four groups of Chinese kale sprouts were then randomly selected from each treatment, with 10 plants per group; the sprouts were weighed to calculate the weight of each plant, and the height of the sprouts was measured with a ruler.

### 2.3. Chlorophyll and Carotenoid Content

Next, 0.04 g dry sample was mixed with 4 mL of 90% acetone extract, ultrasonicated in an ice bath for 20 min, and centrifuged at 4000 *g* at room temperature for 5 min. The supernatant was filtered with a 0.22 μm nylon filter. High-performance liquid chromatography (HPLC) analysis of chlorophyll and carotenoids was performed using an Agilent 1260 instrument and a variable-wavelength detector (VWD) (Agilent Technologies, Inc., Palo Alto, CA, USA). The sample (10 μL) was separated on a Waters C18 column (150 × 3.9 mm i.d.; 4 μm particle size) using isopropanol and 80% acetonitrile–water as the mobile phase. The flow rate was 0.5 mL min^−1^, the column temperature was maintained at 30 °C, and the absorbance was detected at 448 and 428 nm; four biological replicates were used for each treatment [5].

### 2.4. Soluble Sugar

The dried sample (0.02 g) was weighed, placed in a 10 mL centrifuge tube, mixed with 8 mL of distilled water, vortexed for 30 s, and then extracted in a water bath at 90 °C for 20 min. After cooling, the sample was centrifuged at 4000 *g* for 5 min. Then, 1 mL of the supernatant was extracted, 0.1 mL of anthrone–ethyl acetate reagent and 1 mL of concentrated sulfuric acid were added, and then the reaction was carried out in a water bath at 90 °C for 5 min. After cooling, the absorbance at 630 nm was measured within 5 min using four biological replicates per treatment. The soluble sugar content was determined employing the sucrose standard curve [5].

### 2.5. Soluble Protein

Further, 0.02 g of dry sample was weighed and mixed with 8 mL of distilled water. After 40 min, the sample was centrifuged at 4000 *g* for 5 min. Then, 0.25 mL of the supernatant was transferred into a 5 mL centrifuge tube, mixed with 2.5 mL of Coomassie blue dye solution (G-250), and shaken well. The absorbance of the sample was measured at 595 nm after 5 min, using four biological replicates per treatment. The soluble protein content in the sample was calculated employing the standard curve of bovine serum albumin [8].

### 2.6. Ascorbic Acid Content

A 0.04 g aliquot of the sample was extracted with 9 mL of 2% oxalic acid solution for 10 min and then centrifuged at 4000 *g* for 5 min. The supernatant was immediately titrated with 2,6-dichloroindophenol solution to pink and did not fade within 15 s. The volume of 2,6-dichloroindophenol solution consumed was recorded, and the ascorbic acid content was calculated, using four biological replicates per treatment [5].

### 2.7. Flavonoid Content

Next, 0.04 g dry sample was mixed with 3 mL of 50% ethanol, placed at room temperature for 24 h, and centrifuged at 4000 *g* for 5 min. Then, 0.3 mL of the supernatant was taken, mixed with 60 μL of 2% AlCl_3_ solution, 60 μL of 1 mol L^−1^ potassium acetate solution, and 1.68 mL of distilled water, and placed at room temperature for 40 min. The absorbance was measured at 415 nm using an AMR-100 microplate reader (Allsheng Instrument Co., Ltd., Hangzhou, China), with four biological replicates per treatment. The quercetin content was determined employing the standard calibration curve, using the quercetin solution in 50% ethanol as the reference standard [5].

### 2.8. Total Phenolic Content

Further, 0.04 g dry sample was mixed with 3 mL of 50% ethanol, placed at room temperature for 24 h, and centrifuged at 4000 *g* for 5 min. Then, 0.03 mL of the supernatant was taken and mixed with 0.15 mL of Folin–Ciocalteu solution and 0.12 mL of 75% sodium carbonate solution. The absorbance was measured at 760 nm after 1 h using an AMR-100 microplate reader (Allsheng Instrument Co., Ltd., Hangzhou, China), with four biological replicates per treatment. Gallic acid was used as the standard [5].

### 2.9. Ferric Reducing Antioxidant Power (FRAP)

Next, a 0.04 g dry sample was mixed with 3 mL of 50% ethanol, placed at room temperature for 24 h, and centrifuged at 4000 *g* for 5 min. The absorbance was measured at 593 nm using an AMR-100 microplate reader (Allsheng Instrument Co., Ltd., Hangzhou, China), with four biological replicates per treatment. The ferric reducing antioxidant power (FRAP) value was calculated based on the standard calibration curve of FeSO_4_ · 7H_2_O and expressed as μmol g^−1^ dry weight [5].

### 2.10. Composition and Content of Glucosinolates

The dry sample (100 mg) was weighed, placed in a 10 mL centrifuge tube, mixed with 5 mL of distilled water, boiled for 10 min, and centrifuged at 4000 *g* for 5 min to collect the supernatant. The residue was washed with water (5 mL), centrifuged, and combined with the previous extract. The water extract (1 mL) was loaded onto a DEAE-Sephadex A-25 column. The glucosinolates were converted into their desulpho analogs by treating them with 100 μL of 0.1% aryl sulfatase overnight. The desulphoglucosinolates were eluted with 1 mL of water. HPLC analysis of glucosinolates was performed using an Agilent 1260 HPLC instrument equipped with a VWD (Agilent Technologies, Inc., Palo Alto, CA, USA). Acetonitrile and water were used to separate the samples on a Waters Spherisorb C18 column (250 × 4.6 mm i.d.; 5 μm particle size) at 30 °C and a flow rate of 1 mL min^−1^. The absorbance was detected at 226 nm, with four biological replicates per treatment. Glucosinolates were quantified using ortho-nitrophenyl β-D-galactopyranoside as an internal standard and considering the response factor of each glucosinolate [5].

### 2.11. Statistical Analysis

One-way analysis of variance was performed using the SPSS software (version 27, IBM Corporation, Armonk, NY, USA). The least significant difference test was used to compare the means, and the significance level was set at 0.05. The results were expressed as mean ± standard deviation. Microsoft Office 2021 was used to generate charts, SIMCA 14.1 (Umetrics, Malmö, Sweden) was used for principal component analysis (PCA), and Origin 2024 was used for correlation analysis. Four biological replicates were included for each treatment.

## 3. Results

### 3.1. Plant Growth Indicators

**Figure 1:**
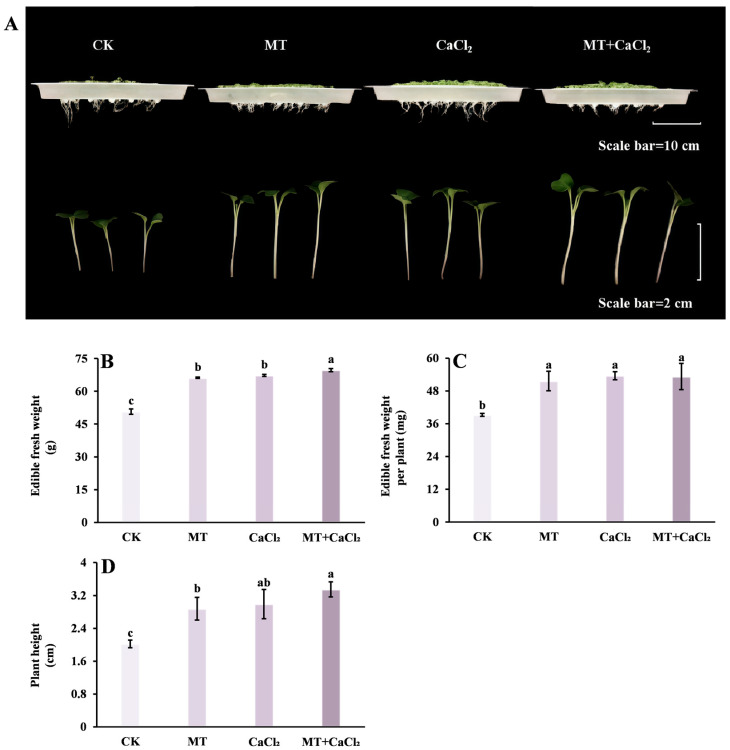
Growth morphology and related physiological indicators of Chinese kale sprouts under different treatments. (**A**) Growth morphology; (**B**) fresh weight; (**C**) fresh weight per plant; and (**D**) plant height. Different letters in the figure indicate statistically significant differences among treatment groups (*p* < 0.05).

Both MT and CaCl_2_ treatments promoted the growth of Chinese kale sprouts, with the combined treatment exerting the greatest effect (Figure 1A). Compared with the control treatment, CaCl_2_ treatment significantly increased the fresh weight, fresh weight per plant, and plant height of Chinese kale sprouts by 32.7%, 36.7%, and 47.8%, respectively, whereas the MT treatment group increased them by 30.5%, 31.7%, and 42.3%, respectively (Figure 1B–D). Moreover, CaCl_2_ and MT treatments significantly enhanced growth performance, with fresh weight and plant height being 1.37 and 1.66 times higher than those under the control treatment, respectively.

### 3.2. Chlorophyll and Carotenoid

**Figure 2:**
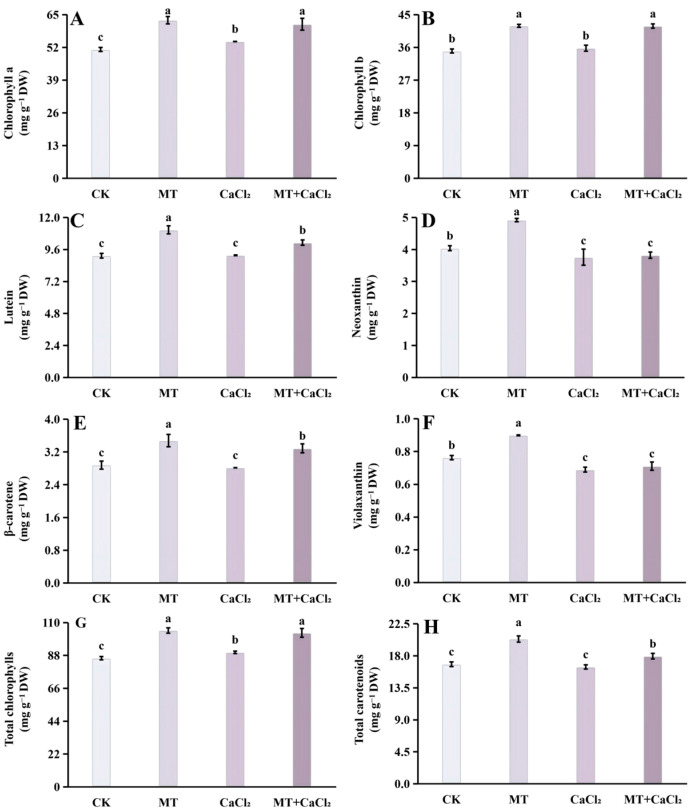
Chlorophyll and carotenoid contents of Chinese kale sprouts under different treatments. (**A**) Chlorophyll a; (**B**) chlorophyll b; (**C**) lutein; (**D**) neoxanthin; (**E**) β-carotene; (**F**) violaxanthin; (**G**) total chlorophylls; and (**H**) total carotenoids. Different letters in the figure indicate statistically significant differences among treatment groups (*p* < 0.05).

The analysis revealed the presence of two chlorophylls and four carotenoids in Chinese kale sprouts (Figure 2A–H). Under different treatments, the sprouts in the MT group exhibited the highest total chlorophyll content, 1.22-fold higher than that in the control group. In contrast, the chlorophyll content under CaCl_2_ treatment was lower than that under MT treatment but still surpassed the control (Figure 2G). The variation trend of chlorophyll a and total chlorophylls was consistent; the chlorophyll a content under the MT treatment was significantly higher than that under control and CaCl_2_ treatments; the content in the compound treatment group was also significantly higher than that in the CaCl_2_ treatment group (Figure 2A). The chlorophyll b content displayed a significant increase under MT treatment compared with that under both control and the CaCl_2_ treatments (Figure 2B).

Among the four carotenoids, the lutein content was the highest, followed by neoxanthin and β-carotene, whereas the violaxanthin content was the lowest (Figure 2C–F). MT treatment significantly enhanced lutein and β-carotene contents, corresponding to increases of 21.2% and 20.7% over the control, respectively (Figure 2C,E). MT also promoted the accumulation of neoxanthin and violaxanthin, with levels 1.22-fold and 1.18-fold higher than that in the control group, respectively, whereas CaCl_2_ and combined treatments reduced their contents (Figure 2D,F). Overall, the MT group exhibited the highest total carotenoid content, which was significantly greater than that under both CaCl_2_ and combined treatments (Figure 2H).

### 3.3. Soluble Sugar and Soluble Protein

**Figure 3:**
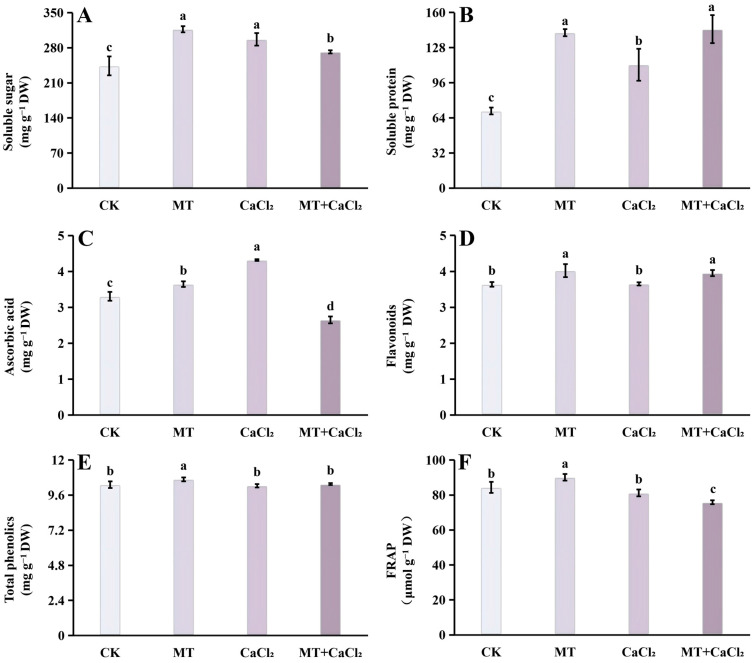
Nutritional quality indices and antioxidant capacity of Chinese kale sprouts under different treatments. (**A**) Soluble sugar content; (**B**) soluble protein content; (**C**) ascorbic acid content; (**D**) flavonoid content; (**E**) total phenolic content; and (**F**) FRAP. Different letters in the figure indicate a statistically significant difference between the treatment groups (*p* < 0.05).

Both MT and CaCl_2_ treatments increased the soluble sugar content of Chinese kale sprouts, with MT showing the greatest effect (30.1% higher than the control), followed by CaCl_2_; however, the combined treatment resulted in lower levels than either single treatment (Figure 3A). The combined and MT treatments were the most effective, each increasing the soluble protein content by approximately twofold, whereas CaCl_2_ treatment alone had a weaker effect, resulting in only a 1.6-fold increase (Figure 3B).

### 3.4. Antioxidant Content and Antioxidant Capacity

MT and CaCl_2_ treatments significantly increased the ascorbic acid content of Chinese kale sprouts compared with the control treatment (Figure 3C). Among these, CaCl_2_ was the most effective, which increased the content by 30.5% compared with the control treatment, followed by MT. However, the combined treatment unexpectedly reduced ascorbic acid content by 19.8% (Figure 3C).

MT treatment significantly increased both flavonoid and total phenolic contents in Chinese kale sprouts (Figure 3D,E). The content under the combined treatment was higher compared with that under CaCl_2_ treatment alone but exhibited no significant difference compared with that under MT treatment. The total phenolic content under the combined treatment was slightly higher than that under CaCl_2_ treatment but remained significantly lower than that under MT treatment. In addition, FRAP reached its maximum under MT treatment, with significant differences among all treatments (Figure 3F).

### 3.5. Glucosinolates

**Figure 4:**
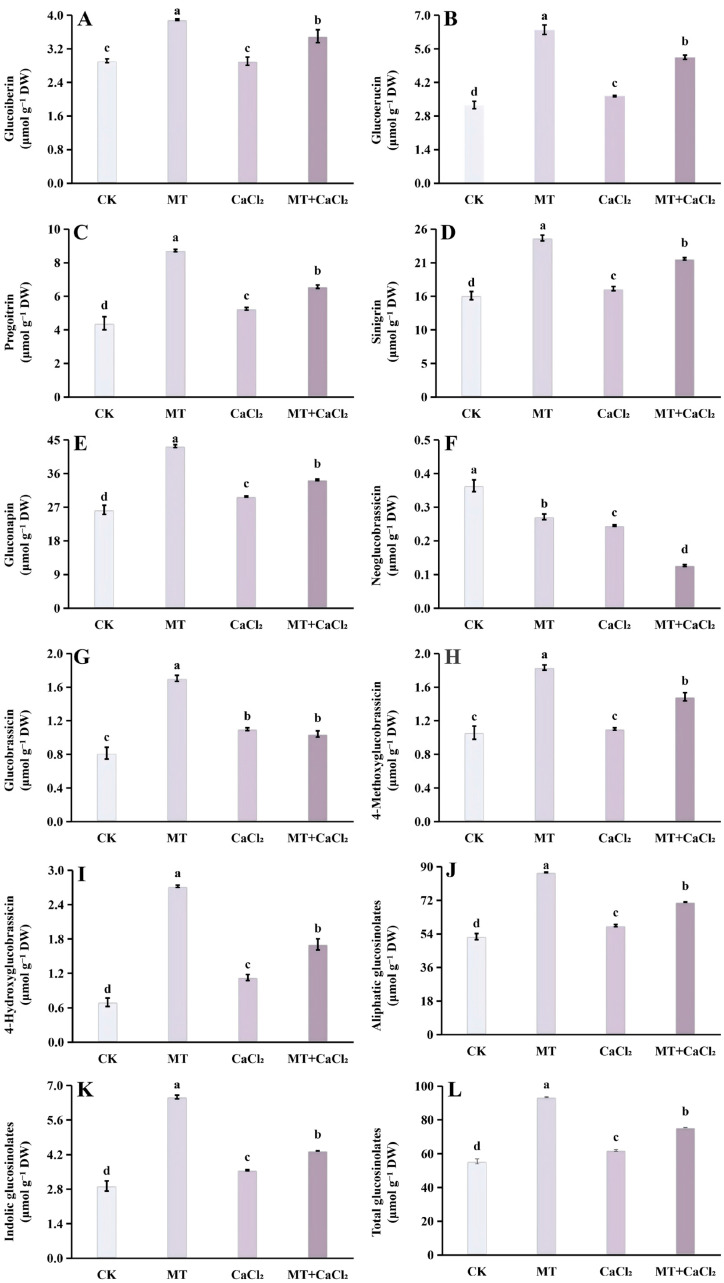
Glucosinolate content of Chinese kale sprouts under different treatments. (**A**) Glucoiberin; (**B**) glucoerucin; (**C**) progoitrin; (**D**) sinigrin; (**E**) gluconapin; (**F**) neoglucobrassicin; (**G**) glucobrassicin; (**H**) 4-methoxyglucobrassicin; (**I**) 4-hydroxyglucobrassicin; (**J**) aliphatic glucosinolates; (**K**) indolic glucosinolates; and (**L**) total glucosinolates. Different letters in the figure indicate statistically significant differences between the treatment groups (*p* < 0.05).

Five aliphatic glucosinolates and four indolic glucosinolates were identified in Chinese kale sprouts (Figure 4A–J). Aliphatic glucosinolates constituted the majority of the total glucosinolate pool. The levels of glucoiberin, glucoerucin, progoitrin, sinigrin, and gluconapin exhibited similar patterns across treatments (Figure 4A–E). All treatments increased aliphatic glucosinolate content compared with the control treatment, with MT treatment causing the highest accumulation, followed by the combined treatment and CaCl_2_ treatment alone. Specifically, MT increased the contents of these five compounds by 1.34-, 1.96-, 1.99-, 1.57-, and 1.65-fold, respectively. In contrast, CaCl_2_ treatment had little effect on glucoiberin content, showing no significant difference compared with the control.

Distinct patterns were observed for indolic glucosinolates. Both MT and CaCl_2_ treatments significantly reduced neoglucobrassicin contents, with the lowest content detected under the combined treatment (0.13 μmol g^−1^ dry weight), representing a 65.2% decrease compared with the control (Figure 4F). In contrast, glucobrassicin accumulation was enhanced by all treatments, with MT yielding the highest content, 2.09-fold greater than that in the control group (Figure 4G). Moreover, MT significantly promoted the accumulation of 4-methoxyglucobrassicin and 4-hydroxyglucobrassicin, reaching 1.73- and 3.90-fold of the control, respectively, followed by the combined and CaCl_2_ treatments (Figure 4H,I).

Overall, the patterns of total aliphatic, total indolic, and total glucosinolate contents were consistent with those of aliphatic glucosinolates: MT treatment resulted in the highest accumulation, followed by the combined and CaCl_2_ treatments, with significant differences among treatments (Figure 4J–L).

### 3.6. PCA

**Figure 5:**
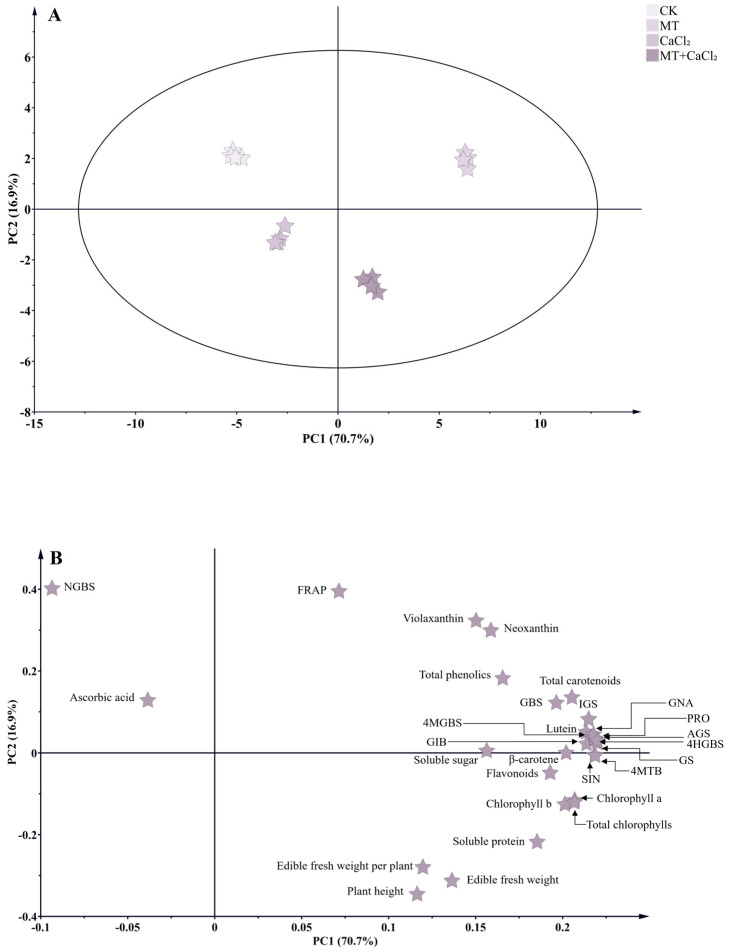
Principal component analysis of Chinese kale sprouts under different treatments. (**A**) PCA score plot; and (**B**) load diagram. GIB, Glucoiberin; 4MTB, glucoerucin; PRO, progoitrin; SIN, sinigrin; GNA, gluconapin; NGBS, neoglucobrassicin; GBS, glucobrassicin; 4MGBS, 4-methoxyglucobrassicin; 4HGBS, 4-hydroxyglucobrassicin; AGS, aliphatic glucosinolates; IGS, indolic glucosinolates; GS, total glucosinolates.

The first and second principal components (PC1 and PC2) explained 70.7% and 16.9% of the total variance, respectively (Figure 5A). PC1 effectively separated the treatment groups from the control group, whereas PC2 further distinguished the MT and CaCl_2_ treatments, indicating clear treatment-specific differences. The loading plot revealed that most growth- and quality-related indicators of Chinese kale sprouts were distributed in the first and fourth quadrants (Figure 5B). Traits strongly associated with MT treatment included the contents of soluble sugar, β-carotene, aliphatic and indolic glucosinolates, total glucosinolates, and total phenolic compounds, as well as neoxanthin and violaxanthin. In contrast, soluble protein content, chlorophyll content, plant height, fresh weight, and fresh weight per plant were clustered in the fourth quadrant, suggesting that these parameters were more strongly influenced by the combined MT and CaCl_2_ treatment.

### 3.7. Correlation Analysis

**Figure 6:**
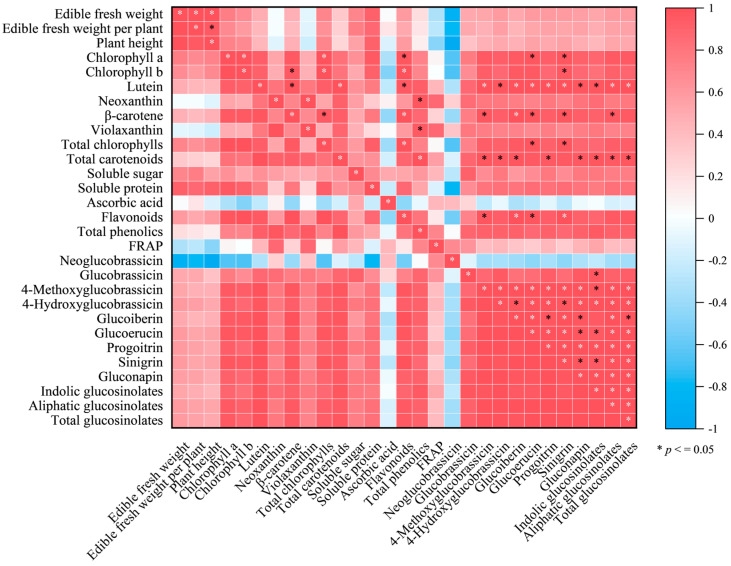
Correlation diagram between compounds and antioxidant capacity in Chinese kale sprouts. All correlations shown represent Pearson correlation coefficients exceeding the threshold (*p* < 0.05).

The correlation analysis (Figure 6) revealed a strong correlation of the edible fresh weight of Chinese kale sprouts with fresh weight per plant (*r* = 0.99) and plant height (*r* = 0.98). Total chlorophyll content also exhibited a strong positive correlation with flavonoid (*r* = 0.98) and sinigrin contents (*r* = 0.96). Similarly, total carotenoid content was positively correlated with total phenolic (*r* = 0.98) and indolic glucosinolate contents (*r* = 0.97). Flavonoid content showed highly significant correlations with sinigrin (*r* = 0.98) and 4-methoxyglucobrassicin contents (*r* = 0.96). In addition, total glucosinolate content was strongly correlated with the contents of multiple compounds, including 4-hydroxyglucobrassicin (*r* = 0.99), glucoiberin (*r* = 0.97), glucobrassicin (*r* = 0.98), progoitrin (*r* = 0.99), and sinigrin (*r* = 0.98) (*p* < 0.05).

## 4. Discussion

Chinese kale is rich in soluble sugar, ascorbic acid, phenolic compounds, glucosinolates, and other healthy ingredients [14]. It is a vegetable with high nutritional value [24]. Numerous studies have demonstrated that exogenous treatments (MT, sucrose, inositol, etc.) that regulate plant physiological and metabolic processes are an effective approach to improving the growth traits and nutritional quality of cruciferous vegetables [25,26]. This study examined the influence of MT and CaCl_2_, applied individually and in combination, on the development and nutritional quality of Chinese kale sprouts.

Fresh weight and plant height directly reflect the yield and economic potential of sprouts, while also serving as indicators of plant vigor and metabolic activity [22]. Both MT and CaCl_2_ treatments promoted sprout growth, with the combined treatment inducing the greatest increase in biomass. The synergistic effect likely results from the complementary roles of MT and Ca^2+^: MT regulates photosynthesis, reduces oxidative stress, and delays senescence [27], whereas Ca^2+^ stabilizes cell walls, enhances membrane integrity, and improves stress resistance [28,29]. The significant increases in fresh weight, plant height, and per-plant biomass suggest enhanced nutrient uptake and accelerated growth under combined treatment.

Chlorophylls and carotenoids are the core components of the photosynthetic system. Their contents not only directly influence the photosynthetic assimilation efficiency of plants but also represent an important class of biologically active compounds. They exert antioxidant and anti-inflammatory effects [30,31]. This study demonstrated that MT treatment markedly enhanced the accumulation of total chlorophylls and carotenoids in Chinese kale sprouts. The mechanism underlying this effect may be related to the ability of MT to increase the synthesis of photosynthetic pigments in Chinese kale, thereby promoting plant photosynthesis and improving the utilization efficiency of light energy by plants [32]. A large number of studies have confirmed that MT can promote chlorophyll accumulation by increasing the supply of precursors for chlorophyll synthesis [33]. At the same time, MT, as a broad-spectrum antioxidant, can effectively alleviate the damage caused by oxidative stress to chloroplast membranes and photosynthetic pigments, reduce chlorophyll degradation, and maintain high pigment contents [34]. MT can also stabilize the thylakoid membrane structure, protect photosystem function, and provide a favorable physiological environment for pigment accumulation and efficient photosynthesis [35]. Similar effects have been reported in many crops. For example, MT can significantly increase the chlorophyll and carotenoid contents in sweet corn, tobacco, pepper, and other crops and enhance photosynthetic efficiency and stress resistance [36].

Soluble sugar and soluble protein are key physiological indicators for evaluating plant carbon and nitrogen metabolism and nutritional quality. Soluble sugars function not only as energy reserves and osmotic regulators but also as signaling molecules involved in signal transduction [37]. Soluble protein content is directly related to nitrogen metabolism and the overall nutritional value of plants [38]. Our results showed that both MT and CaCl_2_ treatments significantly increased the content of soluble sugar in Chinese kale sprouts, with the effect of CaCl_2_ treatment being the most prominent. This result was in line with the findings of Li et al. (2022), indicating that exogenous Ca^2+^ might promote carbohydrate metabolism and sugar accumulation [39]. The effect of MT on sugar content may be indirectly derived from its enhanced carbon metabolism activity, thus providing more precursors for sugar synthesis [40,41,42]. MT treatment significantly increased soluble protein content, which may be due to its activation of nitrogen metabolism and protein synthesis pathways. Existing data indicate that MT treatment can effectively increase the soluble protein content in sand melon, cucumber, and cabbage seedlings by regulating the key processes of nitrogen metabolism and related enzyme activities [43,44,45]. In contrast, the promoting effect of CaCl_2_ treatment alone on protein accumulation was weak, indicating that calcium signaling plays a relatively limited role in regulating the primary nitrogen metabolism pathway [39].

Ascorbic acid is a key intrinsic antioxidant in plants, playing a central role in scavenging ROS and orchestrating cellular redox balance [46]. This study showed that MT and CaCl_2_ treatments individually increased the content of ascorbic acid in Chinese kale sprouts, with the effect of CaCl_2_ treatment being the most significant. The supplementation of Ca^2+^ in lettuce promoted the accumulation of ascorbic acid [27]. The combined treatment of MT and CaCl_2_ did not exhibit a synergistic effect on the accumulation of ascorbic acid; the ascorbic acid content was lower than that under CaCl_2_ treatment alone. This phenomenon is mainly related to the cross-interference of their physiological regulation and the imbalance of resource allocation in plants. Both MT and Ca^2+^ can independently activate the antioxidant system in plants and play a role in stress resistance and protection [47,48]. However, when MT and Ca^2+^ are applied together, their signaling pathways may exhibit cross-interference and mutual antagonism, thereby weakening the regulatory effect observed with a single antioxidant. At the same time, the carbon source, energy, and other nutritional resources in the plant are limited [48]. The combined treatment can guide these resources in the synthesis of flavonoids, phenols, and other antioxidants, rather than their preferential allocation to ascorbic acid, resulting in a decrease in its accumulation.

Flavonoid and total phenolic compounds, as important secondary metabolites in plants, not only have significant antioxidant activity but also serve as key regulators in plant physiological regulation and nutritional quality improvement [49]. In this study, MT treatment markedly enhanced the accumulation of flavonoids and total phenolic compounds in Chinese kale sprouts, consistent with the research findings in other crops. MT treatment in lettuce increased the total phenolic content by more than 25% [4] and significantly improved the antioxidant-related physiological activities of plants. This effect was mainly due to the regulatory effect of MT on the physiological state of plants. It promoted the biosynthesis and accumulation of phenolic compounds [50] while enhancing the supply of reducing power in plants, thereby supporting the stability of phenolic compounds and thus improving the antioxidant capacity and nutritional quality of lettuce [51].

Glucosinolates are unique secondary metabolites of cruciferous plants. On the one hand, they provide vegetables with their distinctive flavor. On the other hand, they serve as key components in plant defense and human health [5]. In this study, MT and CaCl_2_ treatments significantly changed the composition of glucosinolates in Chinese kale sprouts: the content of neoglucobrassicin decreased, whereas the content of other major glucosinolate components increased significantly; MT treatment had the most prominent effect. Further analysis showed that the content of aliphatic glucosinolates increased most significantly under MT treatment. MT could promote the accumulation of this type of glucosinolates by regulating the physiological state of the plant, thereby enhancing the disease resistance and stress resistance of plants [13]. The aforementioned results suggest that MT plays a positive and specific role in regulating glucosinolate metabolism in Chinese kale.

This study explored only the regulatory effects of MT and CaCl_2_ treatments on the bud growth and nutritional quality of Chinese kale from the perspective of plant physiology. It did not address the molecular mechanisms and gene regulatory processes and thus could not explicitly explain the internal regulatory pathways involved. The broader applicability of the findings still requires further verification. However, the results demonstrate strong practical potential for production applications. MT and CaCl_2_ are readily available, low-cost, and easy to apply.

## 5. Conclusions

The study revealed that MT and CaCl_2_ impacted the growth, pigment content, antioxidant content, and glucosinolate accumulation of Chinese kale sprouts differently, either alone or in combination. MT treatment effectively improved the contents of many nutrients in Chinese kale sprouts. CaCl_2_ treatment promoted the accumulation of ascorbic acid in Chinese kale sprouts. The compound treatment increased the growth index of Chinese kale sprouts. In general, MT treatment improved the accumulation of nutrients in Chinese kale sprouts. Also, the increase in glucosinolate content improved the nutritional value of Chinese kale sprouts. In the future, we plan to further explore the interactions between MT and other nutrients, optimize the growth conditions of Chinese kale sprouts, improve plant quality and yield, and promote the accumulation of health-promoting compounds.

## Data Availability

The original contributions presented in the study are included in the article; further inquiries can be directed to the corresponding authors.
